# Bioactive properties of *faveleira* (*Cnidoscolus quercifolius*) seeds, oil and press cake obtained during oilseed processing

**DOI:** 10.1371/journal.pone.0183935

**Published:** 2017-08-28

**Authors:** Penha Patrícia Cabral Ribeiro, Denise Maria de Lima e Silva, Cristiane Fernandes de Assis, Roberta Targino Pinto Correia, Karla Suzanne Florentino da Silva Chaves Damasceno

**Affiliations:** 1 Department of Nutrition, Federal University of Rio Grande do Norte, Natal, Rio Grande do Norte, Brasil; 2 School of Pharmacy, Federal University of Rio Grande do Norte, Natal, Rio Grande do Norte, Brasil; 3 Department of Chemical Engineering, Federal University of Rio Grande do Norte, Natal, Rio Grande do Norte, Brasil; National Cancer Institute at Frederick, UNITED STATES

## Abstract

To the best of our knowledge, this is the first report in the literature concerning the bioactive properties of *faveleira* products. This work focuses on the physicochemical evaluation of *faveleira* oil, as well as it investigates the bioactive properties of *faveleira* seeds, *faveleira* oil and the press cake obtained during the oilseed processing. The seeds were cold pressed and the following tests were performed: physicochemical characteristics (acidity, peroxide values, moisture and volatile matter, density and viscosity) and fatty acid profile of *faveleira* oil; total phenolic and flavonoid content of *faveleira* seed and press cake; antibacterial activity of seed, oil and press cake; and antioxidant activity (DPPH radical scavenging activity, reducing power assay, total antioxidant capacity, superoxide radical scavenging assay and oxygen radical absorbance capacity) of seed, oil and press cake. Our work demonstrated that the *faveleira* seed oil has low acidity (0.78 ± 0.03% oleic acid) and peroxide value (1.13 ± 0.12 mEq/1000g), associated with the relevant concentration of linoleic acid (53.56%). It was observed that important phenolics (398.89 ± 6.34 mg EAG/100 g), especially flavonoids (29.81 ± 0.71 mg RE/g) remain in the press cake, which indicates that the by-product of the *faveleira* oilseed production constitutes a rich residual source of bioactive compounds. No bacterial growth inhibition was detected, but all samples including *faveleira* seeds, press cake, oil and its fractions have potent antioxidant activities, mainly the press cake, with oxygen radical absorbance capacity of 28.39 ± 4.36 μM TE/g. Our results also show that *faveleira* oil has potential to be used as edible oil and the press cake should be used to contain the most antioxidants from seed.

## Introduction

*Caatinga*, also referred as white and/or tropical dry forest, is a semi-arid region of Northeastern Brazil characterized by low annual rainfall, rocky and dry soils and it is one of the most biologically diverse semi-arid regions in the world. Although poorly studied and exploited, several species of the *Caatinga* have been recognized as promising oilseed sources [1].

Among these species, *faveleira* (*Cnidoscolus quercifolius*) is a well-adapted *Euphorbiaceae* plant widely found in the Brazilian *caatinga*. The high quality oil extracted from seeds, of this native plant, can be used for animal feed and for human consumption, besides other uses such as biomass and biodiesel [2, 3]. In fact, *faveleira* seeds have long been consumed by local populations, but their technological potential and industrial applications have not been fully understood and explored yet.

Several oil extraction methods can be used, but the cold pressing is often chosen because it does not use organic solvents or heat in the process, which in turn produces higher quality oils [4]. Also, this technique is considered to be more economical and less labor-intensive than the traditional extraction solvent method [5]. During the oilseed extraction, two fractions are obtained. While apolar and intermediate polarity compounds are transferred to the oil, the polar compounds remain in the press cake, which constitutes the remaining solids after seed pressing [6].

The antioxidant and antibacterial activities of other seeds have been reported [6, 7]. In fact, several phenolic compounds play an important role on these activities, especially flavonoids [8–10]. Despite that, to the best of our knowledge, there are no studies concerning the bioactive compounds of *faveleira* seeds and derived products. Therefore, this work focuses on the physicochemical evaluation of *faveleira* seed oil from the Brazilian *caatinga*, as well as it investigates the bioactive properties of *faveleira* seeds and oil, and the press cake obtained during the oilseed processing.

## Material and methods

### Plant material

The *Cnidoscolus quercifolius* fruits were harvested from São José do Seridó (latitude: 6°26’54”, longitude: 36°52’43”) in Rio Grande do Norte State, Brazil. No specific permissions were required for locations where the seeds were collected. The fruits, all free from injuries, were collected before their dehiscence, between March 2015 and May 2015, in different days and were mixed into a single batch. The seeds were manually extracted from the fruits, totaling 2.5 kg. The specie harvested has been incorporated into the herbarium of UFRN (reference number 20064) and it is not an endangered or protected specie.

### Preparation of extracts

**Fig 1:**
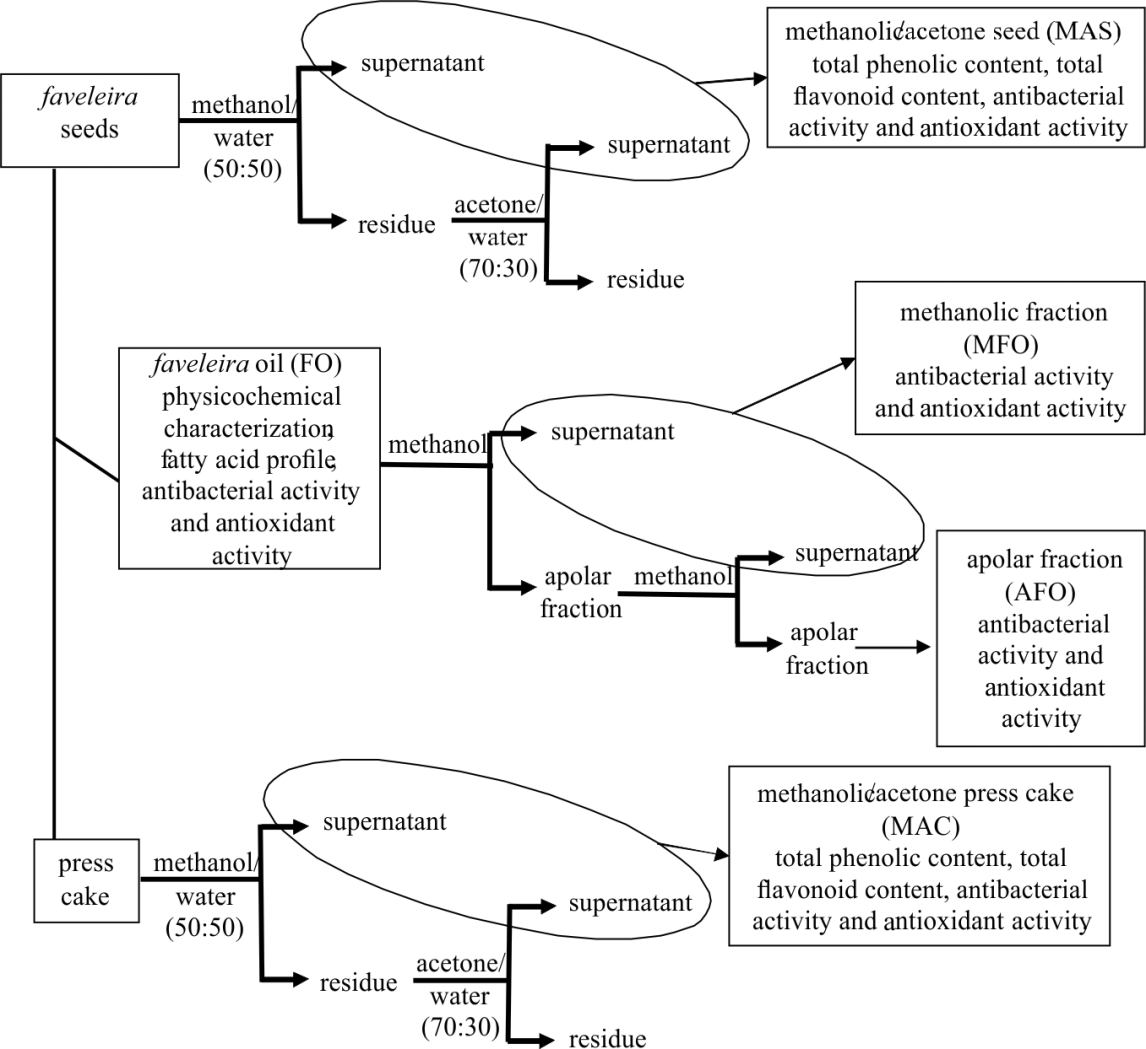
Different fractions obtained from faveleira seeds, oil and press cake and determinations performed.

Fig 1 shows the experimental scheme of the research. Three experimental groups were analyzed: the *faveleira* seeds, the *faveleira* oil and the press cake, a by-product obtained after oilseed extraction. Initially, part of the *faveleira* seeds was used for direct extraction according to Arranz et al. [11]. Briefly, the seeds were ground (Walita, São Paulo, Brazil) and 20 mL of methanol/water 50:50 (v/v) solution was added to 0.5 g of seeds. The mixture was homogenized at room temperature for 1h followed by centrifugation (Fanem, Excelsa 4, 280 R, São Paulo, Brazil) at 20°C for 10 min at 2,500 g. The supernatant was separated and the residue was extracted with 20 mL of acetone/water 70:30 (v/v) solution. The mixture was homogenized again at room temperature for 1h followed by centrifugation at 20°C for 10 min at 2,500 g. Both supernatants were mixed and constitute the methanol/acetone (MAS) seed extract which was analyzed for total phenolic content (TPC), total flavonoids (TF), antibacterial activity and antioxidant activity.

The rest of the seeds were used for oil and press cake production. Initially, the seeds were cold pressed using a hydraulic press (MARCON, MPH-10, Marilia, Brazil). The oil obtained was centrifuged at 20°C for 15 min at 2,500 g and the supernatant constitutes the *faveleira* oil (FO) sample. It was kept frozen at -20°C until further use and it was analyzed for antioxidant and antibacterial activities, physicochemical characterization and lipid profile. The percentage oil yield was calculated: Yield (%) = [(weight of obtained oil after extraction)/(weight of seeds)] x 100. FO was used for subsequent methanolic extractions according to Arranz et al. [11]. For that, 5 mL of FO was mixed with 5 mL of methanol. The mixture was vigorously homogenized for 20 min and centrifuged at 2,500 g for 10 min and the supernatant was recovered. The antioxidant and antibacterial activities were measured directly in the methanolic fraction (MFO) and in the remaining oil (apolar fraction, AFO).

The press cake obtained was used for methanolic/acetone extraction as described previously. The methanolic/acetone extract obtained from the press cake (MAC) was analyzed for total phenolic and flavonoids content, antibacterial activity and antioxidant activity.

### Physicochemical characterization of *faveleira* oil (FO)

The acidity (method Ca 5a-40), peroxide values (method Cd 8–53), moisture and volatile matter (method Ca 2d-25) in the *faveleira* oil were determined using standard AOCS [12] methods. The oil density was evaluated at 25°C using a densimeter (Anton Paar, DMA 4500 M, São Paulo, Brazil). The oil plastic viscosity measurements were conducted at 25°C with a rotational rheometer (Thermo-Scientific, HAAKE MARS, Waltham, EUA). The flow curves were obtained at shear rates varying from 5 to 1010 s^−1^.

### Fatty acid profile of *faveleira* oil

Initially, the fatty acid methyl esters were obtained according to methodology described by Hartman and Lago [13]. They were further analyzed using a GC system [14] (Agilent, 7890A, Santa Clara, USA) equipped with a flame-ionisation detector (FID) and a CP-Sil 88 capillary column (100 m × 0.25 mm i.d., 0.20 μm film thickness, Chrompack, São Paulo, Brazil). During chromatographic analysis, oven temperature was kept at 140°C for 2 min, raised to 235°C at 2.5°C/min and kept for 10 min. The detector temperature was set to 270°C and hydrogen was used as carrier gas (1 mL/min rate). Identification of fatty acid methyl esters (FAMEs) was based on comparison of retention times of standards mixtures. For each sample, the relative FAMEs composition was quantified and data presented as percentage weight for FAMEs composition.

### Total phenolic content (TPC) and total flavonoid content (TF)

The TPC was determined according to Fujita et al. [15] and results were expressed as mg gallic acid equivalents per 100 g of sample (mg GAE/100 g). The TFC was evaluated according to Saravanan and Parimelazhagan [16] as mg of rutin equivalents per 100 g of sample (mg RE/100 g).

### Antibacterial activity

The antibacterial activity was screened according to National Committee for Clinical Laboratory Standards [17]. Nine potentially pathogenic bacterial strains were tested: four Gram-positive (*Staphylococcus aureus* ATCC 29213, *Listeria monocytogenes* ATCC 15313, *Bacillus cereus* ATCC 11778 and *Enterococcus faecalis* ATCC 29212) and five Gram-negative (*Pseudomonas aeroginosa* ATCC 27853, *Enterobacter cloacae* ATCC 13047, *Escherichia coli* ATCC 25922, *Salmonella Typhimurium* ATCC 14028, *Enterobacter aerogenes* ATCC 13048) cultures. The cultures were grown in Muller-Hinton agar for 24 h at 35°C, followed by suspension in sterile saline solution (0.5 of the McFarland scale, 10^8^ CFU/mL). The suspensions were spread on the surface of Muller-Hinton agar plates and 6 mm diameter discs containing 20 μL of sterile extracts. The samples concentration were: MAS (0.25 mg/20 μL), MAC (0.25 mg/20 μL), FO (18.27 mg/20 μL), AFO (19.95 mg/20 μL), MFO (9.14 mg/20 μL).

Positive (amoxilin for Gram-negative cultures and penicillin for Gram-positive cultures) and negative (solvents used in the extraction) controls were also analyzed. The plates were incubated at 37°C for 24 h and the diameters of the inhibition zones were measured using a caliper rule and expressed in millimeters (mm).

### Antioxidant activity

#### DPPH• (2,2-diphenyl-1-picrilhydrazil) radical scavenging activity

It was evaluated by the Nóbrega et al. [18] modified method using 96-well microplates. The absorbance was measured at 517 nm using a spectrophotometer (BioChrom ASYS, UVM 340, Cambridge, UK). A calibration curve was built with concentrations from 30 to 200 μM of Trolox (6-hydroxi-2,5,7,8-tetramethylcroman-2-carboxilic acid). The results were expressed in micromoles of Trolox equivalents per gram of sample (μM TE/g).

#### Reducing power assay

It was evaluated according to a modified procedure based on Wang et al. [19]. Briefly, 200 μL of samples and 100 μL of potassium ferricyanide (1%, w/v) were mixed and incubated at 50°C for 20 min. Afterwards, 180 μL of TCA (10%, w/v), 20 μL of ferric chloride (0.1%, w/v) and 1.5 mL of phosphate buffer (0.2 M, pH 6.6) was added to the mixture. After mixing, the absorbance was measured at 700 nm with a spectrophotometer (Biospectro UV-VIS SP-220, Curitiba, Brazil). Results were expressed as milligrams of ascorbic acid per gram of sample (mg AA/g).

#### Determination of total antioxidant capacity

It was evaluated by the phosphomolybdenum method described by Kumaram and Karunakaran [20] with modifications. Briefly, 100 μL of extracts were combined with 100μL of 4 mM ammonium molybdate/0.6 M sulfuric acid and 100 μL of 28 mM sodium phosphate and 700 μL of distilled water. Distilled water in the place of extract is used as the blank. The tubes containing the reaction solution were incubated at 95°C for 90 min and the absorbance was measured at 695 nm using a spectrophotometer (Biospectro UV-VIS SP-220, Curitiba, Brazil) against blank after cooling to room temperature. The antioxidant activity was expressed as milligrams of ascorbic acid equivalent per gram of sample (mg AA/g) using a standard curve built with different concentrations of AA (25–250 mg/L).

#### Superoxide radical scavenging assay

It was evaluated according to a modified procedure based on Dasgupta and De [21]. An aliquot of 200 μL of sample was mixed with 200 μL of phosphate buffer (50 mM, pH 7.4), 200 μL of metionin 65 mM, 200 μL of EDTA solution 0.5 mM, 200 μL of 0.375 mM Nitrotetrazolium Blue chloride (NBT) and 200 μL of riboflavin 0.5 mM. Control samples were prepared by substituting 200 μL of sample for 200 μL of phosphate buffer (50 mM, pH 7.4). The mixture was incubated under fluorescent light for 15 min and the absorbance read at 560 nm against a blank. The radical scavenging activity (%) was calculated by the absorbance of sample and control ratio: %I = [(A_control_−A_sample_)/A_control_] x 100.

#### Oxygen radical absorbance capacity (ORAC)

The extracts were evaluated according to Ganske and Dell [22] with modifications. Initially, 1 mL of FO and APO were diluted with 1mL of randomly methylated β-cyclodextrin solution 7% (w/v) in acetone:water (1:1) as a solubility enhancer, with subsequent shaking for 10 seconds [23]. In 96-well microplates, 20 μL of diluted extracts were mixed with 120 μL of fluorescein (10 mM of PBS buffer, pH 7.4). The microplates were incubated for 10 min at 37°C and 60 μL of 2,2'-azobis (2-amidinopropane) dihydrochloride (AAPH) 10.85 g/L in PBS buffer was added. The fluorescence intensity (485 nm excitation and 528 nm emission) was assessed using a spectrophotometer (BMG LABTECH, Fluostar Optima, Ortenberg, Germany) every 3 min up to 180 min. The results were expressed in micromoles of Trolox equivalents per gram of sample (μM TE)/g).

### Statistical analyses

Except for the lipid profile, all samples were analyzed in triplicate and all data were expressed as means and standard deviation (SD). Results were tested for normality using the Shapiro-Wilk test. Statistical significance was evaluated by ANOVA and t test (p<0.05) with the software Statistica 7.0 (StatSoft, Tulsa, USA).

## Results and discussion

### Physicochemical properties of *faveleira* seed oil

The *faveleira* oil extraction yield was 13.9%, which was higher that stabilized baru seed oil extraction (7.99%) [24], but inferior to pecan nut extraction (51%) conducted by Prado et al. [25].

Table 1 shows results from physicochemical properties of *faveleira* oil analysis. The acidity and peroxide values were inferior than *faveleira* (4.33% in oleic acid and 6.99 mEq/1000g, respectively) [26] and hemp seed oils (0.89% in oleic acid and 1.94 mEq/1000g, respectively) [27] previously reported. These parameters are indicators of hydrolytic and oxidative rancidity and lower values indicate better quality oil.

**Table 1:** Physicochemical characterization of *faveleira* seed oil.

	Mean ± SD
**Acidity (% oleic acid)**	0.78 ± 0.04
**Peroxide values (meq/1000g)**	1.13 ± 0.12
**Moisture and volatile matter (%)**	0.25 ± 0.02
**Density (g/cm**^**3**^**)**	0.91 ± 0.00
**Viscosity (Pa.s)**	0.05 ± 0.00

The percentage of moisture and volatile matter of *faveleira* oil was lower than hemp (0.78%), flax (0.60%) and canola (0.65%) seed oils [27]. Low moisture and volatile matter are desirable and indicate higher resistance to decomposition. Higher moisture may lead to the accelerated formation of free fatty acids, which decrease the oil quality [28]. The density of *faveleira* oil in this study was very similar to rubber seed oil (0.92 g/cm^3^) [29] and its viscosity was close to canola oil (0.05 Pa.s) [30].

Several physicochemical characteristics of *faveleira* oil encourage its use as edible oil. Features such as low moisture, acidity and free fatty acid content, associated with desirable color, flavor and resistance to degradation are some of the key features that justify further technological and commercial exploration of this vegetable oil [31].

### Fatty acid profile of *faveleira* seed oil

Unsaturated fatty acids, mainly polyunsaturated, were predominant in *faveleira* oil (Table 2). The most abundant fatty acid found was linoleic acid (53.56%), followed by oleic acid (17.78%). Similar results were observed by Medeiros et al. [26] and Santos et al. [31] in *faveleira* oil. Linoleic acid is the most abundant fatty acid of well-established vegetable oils found in the market such as soy, sunflower and corn oils [30].

**Table 2:** Lipid profile of *faveleira* seed oil.

Fatty acid	%
**Myristic (14:0)**	0.30
**Palmitic (16:0)**	17.55
**Margaric (17:0)**	0.08
**Stearic (18:0)**	9.24
**Arachidic (20:0)**	0.41
**Oleic (18:1)**	17.78
**Cis-11-eicosenoic (20:1 *cis11*)**	0.19
**Linoleic (18:2)**	53.56
**Alpha linolenic (18:3 α)**	0.89
**Saturated**	27.58
**Unsaturated**	72.42
**Monounsaturated**	17.98
**Polyunsaturated**	54.44

Unsaturated fatty acids have long been recognized by their high nutritional value and health relevant effects. For example, linoleic acid has proved to exert hypolipidemic and hepatoprotective roles [32], while oleic acid has shown cardioprotective effects [33].

The ratio of omega-6 to omega-3 essential fatty acids (ω-6/ω-3) of *faveleira* oilseed (60.18) was similar to corn oil (64.15), but lower than gingelly oil (102.24) and cotton seeds (146.7) [34]. The Food and Agriculture Organization (FAO) and the World Health Organization (WHO) recommends a ω-6/ω-3 ratio between 5:1 and 10:1 [35], but it has been claimed that the current western diet is characterized by ω-6/ω-3 ratio of 20:1 [36]. The faveleira oilseed is rich in ω-6 essential fatty acids and its consumption should be associated with increased ingestion of ω-3 rich foods.

### Total phenolic and flavonoids content of *faveleira* products

Results show that the total phenolics and flavonoids of *faveleira* press cake (398.89 mg EAG/100 g and 29.81 mg RE/g, respectively) was substantially higher (p = 0.0002) than *faveleira* seed (324.92 mg EAG/100 g and 18.70 mg RE/g). Despite that, the *faveleira* seed has higher TPC when compared to several other oilseeds such as almonds, Brazil nut, cashew nut, macadamia and pinion which have TPC ranging from 22.5 to 86.7 mg EAG/100 g [37].

The concentration of phenolic compounds in the press cake is due to the fact that several phenolic compounds are polar in nature, and are preferentially retained in it. Similar behavior was observed by Matthaüs [38] and Slatnar et al. [39] after analyzing the TPC of several press cakes obtained from different raw materials. In addition, the Folin-Ciocalteau reagent is more efficient in polar systems, which might play a role on the higher TPC detected in the press cake [25].

Flavonoids were lower quantities in tarap seed (3.65 mg RE/g) when compared to *faveleira* [40]. Previously, Sobrinho et al. [41] found lower flavonoid concentrations in *faveleira* barks (0.6 mg RE/g) and leaves (26.51 mg RE/g). These results show that the *faveleira* press cake is a rich phenolic source and it could be used as an inexpensive and innovative source of natural phytochemicals [42].

### Antibacterial activity

No *faveleira* extracts were able to inhibit the growth *Staphylococcus aureus*, *Listeria monocytogenes*, *Bacillus cereus*, *Enterococcus faecalis* (Gram-positive) and *Pseudomonas aeroginosa*, *Enterobacter cloacae*, *Escherichia coli*, *Salmonella Typhimurium*, *Enterobacter aerogenes* (Gram-negative). Previously, Paredes et al. [43] have shown that methanolic extracts of *faveleira* leaves, bark and root bark showed some inhibition of *Enterococcus faecalis* and *Pseudomonas aeruginosa*, but no activity against *E*. *coli* was observed. In fact, the antibacterial activity of plant extracts is closely associated with the extraction procedure. Depending on the type of solvent used, various compounds with different polarities and concentrations are obtained at the end of the process [44].

### Antioxidant activity

Table 3 shows the antioxidant activity of *faveleira* seeds (MAS) and press cake (MAC) extracts. Overall, the *faveleira* press cake had more potent antioxidant activity when compared to the seeds. Indeed, higher results were observed for reducing power assay (p = 0.0000008), total antioxidant activity (p = 0.0023), ORAC (p = 0.0015) and DPPH (p = 0.0011) assays. This higher antioxidant power correlates well with MAC higher phenolic and flavonoid contents previously shown [45]. Superoxide scavenging results of *faveleira* seeds and press cake were lower than rice seeds cultivars in India (43–79%) [46], but ORAC results were higher than peanut (4.3–7.3 μM TE/g) [47].

**Table 3:** Antioxidant activity of seed and press cake.

	MAS	MAC
	(mean ± SD)	(mean ± SD)
**DPPH radical scavenging activity (μM TE/g)**	7.31 ± 0.05^b^	8.31 ± 0.20^a^
**Reducing power assay (mg AA/g)**	13.67 ± 0.15^b^	22.96 ± 0.27^a^
**Total antioxidant capacity (mg AA/g)**	1.55 ± 0.29^b^	2.79 ± 0.11^a^
**Superoxide radical scavenging assay (%)**	21.86 ± 2.85^a^	22.67 ± 1.80^a^
**ORAC (μM TE/g)**	23.40 ± 0.50^b^	28.08 ± 0.91^a^

MAS, methanol/acetone seed; MAC, methanol/acetone press cake. Different lowercase letters (a, b) show differences between the samples (t test, p<0.05).

In contrast, different responses were found when analyzing the antioxidant activity of *faveleira* oil (FO) and its fractions (MFO and AFO) (Table 4). In fact, only FO and AFO results were statistically compared, since MFO is a methanolic fraction and the solvent might interfere on the results [48]. The crude oil (FO) had higher results for total antioxidant capacity (p = 0.0034) and DPPH assay (p = 0.0015) when compared to AFO. On the other hand, the residual oil (AFO) had higher superoxide scavenging (p = 0.0014) and ORAC (p = 0.0002) results.

**Table 4:** Antioxidant activity of *faveleira* seed oil.

	FO	AFO	MFO
	(mean ± SD)	(mean ± SD)	(mean ± SD)
**DPPH radical scavenging activity (μM TE/g)**	2.35 ± 0.07^a^	1.94 ± 0.06^b^	1.08 ± 0.01
**Reducing power assay (mg AA/g)**	0.11 ± 0.00^a^	0.11 ± 0.00^a^	1.59 ± 0.03
**Total antioxidant capacity (mg AA/g)**	0.04 ± 0.00^a^	0.02 ± 0.00^b^	0.08 ± 0.00
**Superoxide radical scavenging assay (%)**	20.12 ± 2.87^b^	44.72 ± 6.34^a^	13.06 ± 3.47
**ORAC (μM TE/g)**	1.57 ± 0.20^b^	3.30 ± 0.07^a^	1.24 ± 0.11

FO, *faveleira* oil; MFO, methanolic fraction; AFO, apolar fraction. Different lowercase (a, b) letters show differences between the samples (t test, p<0.05).

Such differences are justified by the complex composition of vegetable oils and by the particularities of each analytical method [49, 50]. It has been recommended the use of multiple methods for the assessment of antioxidant activity since they vary in terms of substrates, mechanisms, reactions conditions and results expression [51]. For example, they can be based on the radical scavenging ability such as the DPPH method and the superoxide radical scavenging assay [52]. On the other hand, some methods express the metal reducing power of the extracts, such as the reducing power assay and the total antioxidant capacity [53]. The ORAC method is another widely used protocol and it measures the antioxidant inhibition of peroxyl-radical-induced oxidations [22].

Interestingly, for most of antioxidant assays, the sum of the antioxidant activities of the oil fractions MFO (polar) and AFO (apolar) was higher than FO alone. In FO samples, all compounds were mixed together and it might lead to antagonist effects. Similar response was observed for DPPH results of olive oil [54]. In addition, higher ORAC results were found for AFO samples, which indicate that faveleira apolar compounds were effective in protecting from oxidative damage. It was the use of RMCD that assure the dissolution of apolar components in the reagents used during the assay.

The walnut oil has lower DPPH scavenging activity (0.47 μM TE/g) [55] than faveleira oil, but FO had lower total antioxidant activity than moringa seed oil (37.94 mg AAE/g) [56]. In addition, the sesame oil extracted by cold pressing had higher ORAC results 230.22 μM TE/g) [23] when compared to faveleira oil.

## Conclusions

To the best of our knowledge, this is the first report in the literature showing the bioactive properties of *faveleira* oilseed production. *Faveleira* seeds, press cake, oil and its fractions have potent antioxidant activities and the by-product of the *faveleira* oil production constitutes a rich source of phenolics, specially flavonoids. Here it was demonstrated that the faveleira oilseed has desirable physicochemical properties which might justify its use as edible oil. Despite that, further toxicological studies should be performed to confirm this potential use. This work encourages a deeper study on these bioactive properties of seed and its derivatives and encourages their application as functional foods or as ingredients that increase the bioactive potential of foods.

## Supporting information

S1 Dataset The raw data for Tables 1, 3 and 4. (XLSX)
