# Correction: Bioactive properties of *faveleira* (*Cnidoscolus quercifolius*) seeds, oil and press cake obtained during oilseed processing

**DOI:** 10.1371/journal.pone.0189563

**Published:** 2017-12-07

**Authors:** The PLOS ONE Staff

The following information is missing from the Funding section: This work was supported by the Postgraduate Pro-rectory of the Federal University of Rio Grande do Norte, Postgraduate Program in Nutrition of the Federal University of Rio Grande do Norte (PPGNUT), and Improvement Coordination of Higher Level Personnel (Social Demand/CAPES that provides the scholarship, and FAPERN/CAPES 006/2014 –that provides for "SUPPORT TO THE POSTGRADUATE PROGRAMS OF HIGHER EDUCATION INSTITUTIONS IN THE STATE OF RIO GRANDE DO NORTE”). The funders had no role in study design, data collection and analysis, decision to publish, or preparation of the manuscript.

The publisher apologizes for the error.
